# Focal osteoporotic bone marrow defect involving dental implant: a case report

**DOI:** 10.1186/s40729-015-0020-4

**Published:** 2015-07-17

**Authors:** Natália Galvão Garcia, Francisco Barbara Abreu Barros, Márcia Maria Dalmolin Carvalho, Denise Tostes Oliveira

**Affiliations:** 1 Department of Stomatology, Area of Pathology, Bauru School of Dentistry, University of São Paulo, Bauru, São Paulo Brazil; 2 Dentist, Private Practice, Campinas, São Paulo Brazil

**Keywords:** Bone marrow, Defect, Dental implant

## Abstract

In oral implantology, the most serious complications occur intraoperatively or within a short period. We describe an unusual case of focal osteoporotic bone marrow defect involving dental implant in the posterior mandibular region of the adult woman. Despite the fact that this condition requires no treatment, it could lead to the displacement of the dental implant. Additionally, this case report reinforces that histopathological analysis is mandatory for precise diagnosis of the radiolucency into posterior mandibular region of the adult woman associated or not with dental implant placement.

## Background

In oral implantology, the most serious complications and those most frequently described in the literature occur during surgery [1]. The well-known intraoperative complications and accidents related to surgery have included hemorrhage, nerve damage, mandibular fractures, damage to adjacent teeth, lack of primary stability, and displacement or migration of implants [2].

The displacement of an implant will occur intraoperatively or within a short period because of poor surgical technique or anatomic variances [2]. In addition, the presence of the medullar component in the jawbones causing the focal osteoporotic bone marrow defect can facilitate the displacement of dental implants during surgery [2, 3].

The focal osteoporotic bone marrow defect corresponds to the uncommon hematopoietic tissue found mainly in posterior mandibular region of the adult woman [2, 4, 5]. This condition is generally asymptomatic and discovered during radiographic exam of the jaws [2, 4]. Most of the focal osteoporotic bone marrow defects occur in edentulous areas region where tooth extraction was previously performed [5]. Recently, Sençimen et al. (2011) reported a case of the focal osteoporotic bone marrow occurring secondary to dental implant placement [6].

This paper describes an unusual case of focal osteoporotic bone marrow defect involving dental implant in posterior mandibular region of the adult woman.

## Case presentation

**Fig. 1:**
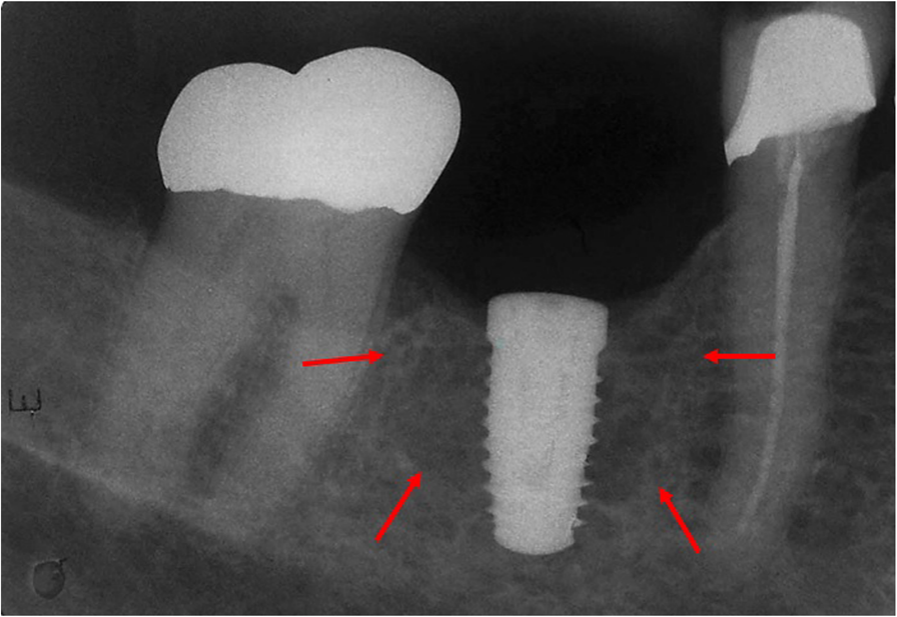
The periapical radiograph revealed the presence of an extensive and poorly circumscribed osteoporotic area around the proximal implant

**Fig. 2:**
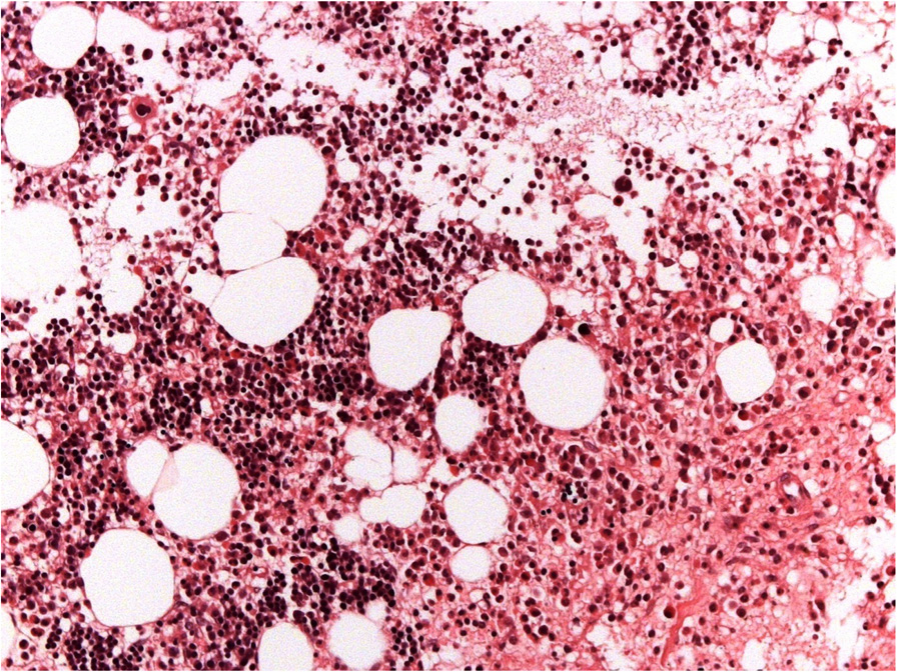
Normal hematopoietic cells, fat cells and bone trabeculae (hematoxylin and eosin, original magnification ×200)

**Fig. 3:**
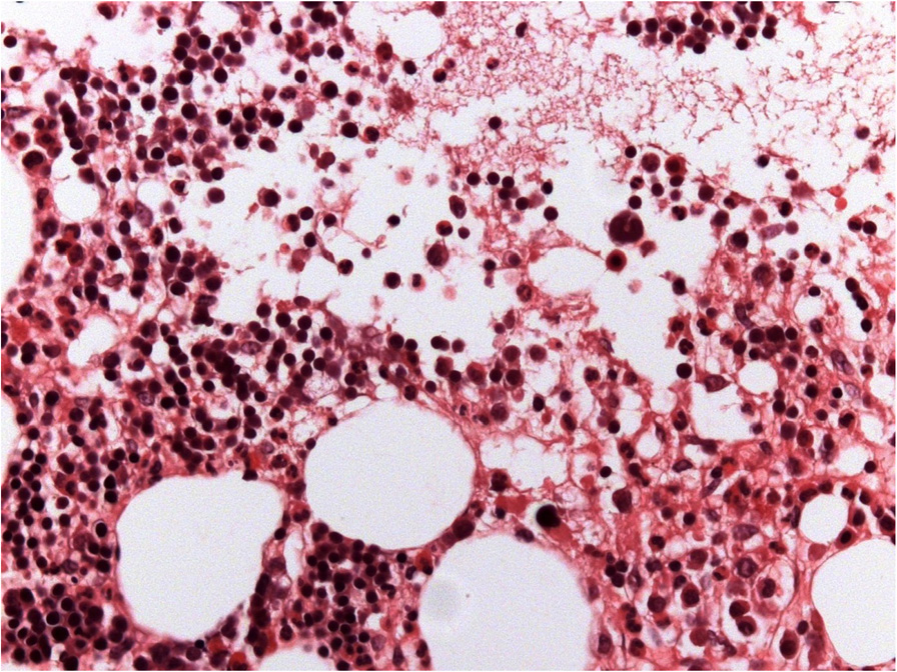
Erythroid, granulocytic, monocytic and lymphocytic series are illustrated, as well as megakaryocytes (hematoxylin and eosin, original magnification ×400)

An 84-year-old white woman was referred to private dental clinic for an implant rehabilitation treatment. Intraoral examination revealed healthy mucosa and there was not any sign of infection. Her past medical history was unremarkable. The radiography of the molar region showed with a quite ill-defined radiolucent area presenting irregular borders associated to dental implant (Fig. 1). This condition was asymptomatic and no expansion of the mandibular cortical bone was detected. Under local anesthesia, a biopsy was performed and the tissue submitted to the Bauru School of Dentistry Oral Pathology Biopsy Service of the University of São Paulo. The histopathological examination revealed normal hematopoietic bone marrow characterized by erythroid, granulocytic, monocytic, and lymphocytic series (Fig. 2). In addition, megakaryocytes, fat cells, and bone trabeculae were also observed (Fig. 3). Abnormal morphology of the hematopoietic cells or malignant cells was not seen. The histopathological findings confirmed the diagnosis of focal osteoporotic bone marrow defect of the mandible.

## Discussion

Focal osteoporotic bone marrow defect is a condition that corresponds to the uncommon presence of hematopoietic tissue found in edentulous areas of the jaws in middle-age women [6].

Clinically, the focal osteoporotic bone marrow defect occurs as isolated or multifocal radiolucency with several millimeters to centimeters in diameter and ill-defined borders [4]. This condition is rarely mentioned in differential diagnosis of radiolucent lesions of the jaws, and the radiographic appearance may be confused with other intraosseous pathologic conditions such as osteomyelitis, traumatic bone cyst, and fibrous dysplasia [4, 6].

The exact cause of focal osteoporotic bone marrow defect is unknown but failure in bone repair in areas of trauma has been associated with its occurrence in edentulous region where tooth extraction [4, 5] or dental implant [3, 6] such as our case report, was previously performed. Sençimen et al. (2011) suggested that the proliferation of hematopoietic marrow elements around the dental implant may be a response of the healing spongious bone to surgically initiated trauma which may occur due to overheating of bone or peri-implant bone compression at the implant osteotomy site by drilling a narrower osteotomy [6].

Only few cases of the focal osteoporotic bone marrow defect associated with dental implants has been described in the English literature [3, 6]. Sençimen et al. (2011) reported a clinical case in which the focal osteoporotic bone marrow defect occurred 2 years postoperatively apical to a dental implant in posterior mandible region and the diagnosis was established based on the combination of clinical, radiographic, and microscopic features [6]. Posteriorly, Lee et al. (2013) reported three clinical cases of displacement of dental implants into the focal osteoporotic bone marrow defect, but the final diagnoses of this condition were based only on clinical and radiographic findings [3]. In this paper, we described a case of the focal osteoporotic bone marrow defect associated to dental implant which was diagnosed by histopathological analysis.

Microscopically, the presence of hematopoietic marrow composed of monocytic, erythroid, granulocytic, and lymphocytic series as well as megakaryocytes associated with fatty marrow is required for diagnosis of focal osteoporotic bone marrow defect [3, 4].

In the present case the final diagnosis was established based on the combination of radiographic and microscopic features. Some authors have based the focal osteoporotic bone marrow defect diagnosis on age, site, and clinical and radiographic findings [3], but it is important to reinforce that the final diagnosis of this condition should be established on microscopic features rather than clinical and radiographic parameters [4].

## Conclusions

This is the second well-documented case report including clinical, radiographic, and microscopic analysis of focal osteoporotic bone marrow defect involving dental implant. Despite the fact that this condition requires no treatment, it could lead to the displacement of the dental implant. In addition, we would like to alert that histopathological analysis is mandatory for precise diagnosis of the radiolucency into posterior mandibular region of the adult woman associated or no with dental implant placement.

## Consent

Written informed consent was obtained from the patient for publication of this case report and any accompanying images. A copy of the written consent is available for review by the Editor-in-Chief of this journal.
